# Student to midwife transition: Newly qualified midwives’ experiences in Limpopo province

**DOI:** 10.4102/hsag.v27i0.1992

**Published:** 2022-11-14

**Authors:** Khathutshelo G. Simane-Netshisaulu

**Affiliations:** 1 Department of Advanced Nursing Science, Faculty of Health Sciences, University of Venda, Thohoyandou, South Africa

**Keywords:** experience, midwifery services, newly qualified midwife, student nurse, transition

## Abstract

**Background:**

Regardless of educational pathway, the transition from student to the registered midwife is a topic of increasing interest within the profession as this is likely to have implications for retention and attrition. Newly qualified midwives have reported that the reality of putting knowledge into practice in the midwifery field is often challenging and stressful.

**Aim:**

To explore and describe the experiences of newly qualified midwives with regard to the provision of midwifery services during transition from students to qualified midwives, in the Limpopo province, South Africa.

**Setting:**

The study was conducted in the maternity units of each of the five selected public hospitals in the Limpopo province.

**Methods:**

The researcher employed a qualitative approach with explorative and descriptive design. The population comprised all midwifery graduates working in the Limpopo province and have undergone a comprehensive nursing programme regulated by R425 of 19 February 1985, as amended. Five newly qualified midwifery graduates were sampled from each of the five selected hospital, using a non-probability purposive sampling method, resulting in a sample size of 25 participants. Data collection was carried out through unstructured individual interviews and was conducted until participants were no longer bringing new information.

**Results:**

Graduates reported excessive workload, which resulted in physical exhaustion. Challenges related to the roles and responsibilities of a new position as a qualified midwife were expressed. Negative collegial relationship displayed by experienced qualified midwives, negatively affected graduates’ midwifery performance.

**Conclusion:**

Effective, transition from student to qualified midwife is a stressful and exhausting process because of unfavourable working conditions in the labour ward, administrative roles as well as negative collegial relationship and disrespect displayed by experienced midwives.

**Contribution:**

Strategies to effectively support newly qualified midwives may be developed, which may consequently help in improving newly qualified midwives’ performance.

## Introduction

Institutions of higher education and training effectively prepare midwifery students to ensure graduates are equipped to deliver quality midwifery care (Royal College of Midwives 2014).

Although newly qualified midwives have successfully completed their training, they may not be competent and confident concerning the performance of certain skills (Black 2018; Ngcobo, Baloyi & Jarvis 2021). Newly qualified midwives find it difficult to perform as professional nurses because they are still insecure (Duchscher 2009; Ngcobo et al. 2021). Experiences of newly qualified midwives during the initial period of placement following completion of training affect their performance level (Kitson-Reynolds, Ferns & Trenerry 2015).

In a study conducted by Kensington et al. (2016), the process of transition from being a midwifery student to a qualified midwife was reported as critical, challenging and stressful as graduates undergo significant role adjustments while experiencing mixed emotions. Mixed emotions result not only from the excitement of successful completion of training but also fear that they may not cope with the demands of a qualified midwife’s role (Kensington et al. 2016).

Lewis and McGowan (2015) described the transition process from being students to becoming qualified midwives as exciting, yet graduates experience shock when they discover that there is a great difference between the role of being a student and that of a qualified midwife. In Ireland, newly qualified midwives experienced frustration and reality shock as they failed to meet the expected level of performance in the clinical practice (Van Der Putten 2008). Newly qualified midwives found it difficult to progress from being students to becoming qualified midwives because of lack of confidence, resulting in anxiety (Duchscher 2009).

The same sentiments were echoed by Wain (2017) who revealed that newly qualified midwives become confused when faced with the reality of practice realising that they fail to cope with the increased demands of a qualified midwife’s role, as they perceived themselves as highly knowledgeable. The author further revealed that midwifery graduates expressed anxiety and insecurity as they crossed over from the protective stance of being students to becoming accountable midwives (Wain 2017). According to Dixon et al. (2015), practicing midwives had unrealistic expectations from newly qualified midwives, as they expected them to adapt quickly to the clinical setting. Newly graduated midwives experienced physical responses to transition shock, which were presented with somatic complaints such as headaches (Odland, Sneltvedt & Sörlie 2014; Reynolds, Cluett & Le-May 2014). Midwifery graduates were overwhelmed and felt inadequate as they were not accepted as part of the team and, therefore, denied the opportunity to learn, which negatively affected their competence and confidence level predisposing them to vulnerability (Black 2018; Griffiths et al. 2019).

*South African Nursing Act* (33 of 2005) dictates that newly qualified midwives whose training is regulated by R425 (February 1985 as amended) are placed in clinical state healthcare facilities to implement a community service programme for a period of 12 months *(Nursing Act* 33 of 2005; South African Nursing Council [SANC] 1992).

The reason why newly qualified midwives are placed in the state healthcare facilities is that they should receive the necessary assistance from experienced and qualified midwives, which may enable them to effectively progress from being students to becoming qualified midwives who are autonomous, competent and effective professionals capable of provision of quality midwifery care. At the end of community service programme, newly qualified midwives are registered with the SANC as independent practitioners (Simane-Netshisaulu & Maputle 2021).

Newly qualified midwives reported a need for support during transition, as problems related to negative behaviour displayed by senior colleagues and inadequate material resources still existed (Ndaba 2013). In a study conducted in the Limpopo province by Simane-Netshisaulu and Maputle (2021), it was reported that despite community service programme prescribed by the SANC, newly qualified midwives were found assisting patients giving birth with no supervision from experienced midwives. In some situations, newly qualified midwives were allocated to be in charge of running shifts, which is not expected because they are to function under the supervision of an experienced midwife, especially during the initial period of community service programme.

The aim of the study was to explore and describe newly qualified midwives’ experiences with regard to the provision of midwifery services during transition from students to qualified midwives, in the Limpopo province.

## Methods

### Study setting

The Limpopo province comprises five districts, namely Vhembe, Mopani, Capricorn, Sekhukhune and Waterberg. One tertiary hospital was selected from each of the five districts (Vhembe, Mopani, Capricorn, Sekhukhune and Waterberg). There are seven district hospitals in Vhembe, six district hospitals in Mopani, five district hospitals in Capricorn, six district hospitals in Sekhukhune and eight district hospitals in Waterberg. The tertiary hospitals were selected by virtue of being referral hospitals with high number of patients and high number of newly qualified midwives. The study was conducted in maternity unit of each of the five selected tertiary hospitals. A maternity unit is a unit that is composed of four sections: ante-natal care unit, labour unit, post-natal unit and neonatal unit. However, post-natal is divided into two sub-sections: for women who delivered normally and for those who delivered through caesarean section.

### Design

A qualitative research approach that was explorative and descriptive in nature was conducted to enable the researcher to fully understand the experiences of newly qualified midwives with regard to provision of midwifery services as they transit from being students to qualified midwives.

### Population and sampling

The population was composed of all newly qualified midwives who completed the training regulated by (R425 of 19 February 1985, as amended) the universities as well as college of nursing and were qualified as general nurses with psychiatry, community health and midwifery working in selected hospitals in the Limpopo province, South Africa. Non-probability purposive sampling strategy (Etikan & Bala 2017) was used to select five newly qualified midwives from every selected facility. The sampling method was used to enable the researcher to select participants who were knowledgeable regarding the phenomenon under study, so that full, relevant and in-depth information could be obtained. Five newly qualified midwives were selected from each hospital so that all the sub-sections in maternity unit might be represented. There are four sub-sections; however, post-natal women have two categories: those who delivered normally and those who delivered through caesarean section. Newly qualified midwives who participated in the study were those whose practicing period after completing the training was not more than 12 months and were placed in maternity units in the facilities sampled for the study. Sample size was 25 newly qualified midwives, of which five participants were selected from every chosen hospital.

### Data collection

In-depth interviews were used to gather data that unstructured individual interviews were used to gain an in-depth understanding of newly qualified midwives’ experiences regarding provision of midwifery services during transition from student to qualified midwife in the Limpopo province, South Africa. The interview was guided by a central question: ‘What experiences do you have with regard to working as a midwife when progressing from being a student to becoming a qualified midwife?’ Probing was performed to encourage the participants to deepen the responses (Creswell 2016). Non-verbal cues and field notes were observed during the interviews.

The researcher spent about an hour interviewing each participant, until data saturation was reached after interviewing 22 participants; however, 3 more participants were interviewed to confirm saturation of data. Data saturation occurs when no more new information is obtained from participants during interviews (De Vos et al. 2013). As a result, a total of 25 participants were interviewed.

### Data analysis

The researcher analysed and coded data based on Tesch’s open-coding method by Creswell (2016) as follows: All the recordings were transcribed verbatim by the researcher. The researcher carefully read through all the transcripts to get a sense of a whole. When all transcripts were concluded, the same topics were listed. Grouping of data was carried out based on themes as well as sub-themes. Coding and categorisation of field notes was performed. The findings were contextualised by the use of literature.

### Trustworthiness

Principles to ensure credibility, transferability, dependability and confirmability were observed as measures to ensure trustworthiness (De Vos et al. 2013).

Credibility was ensured by prolonged engagement with participants to establish rapport and build trust. The researcher spent about an hour with participants conducting interviews listening to them and observing their gestures. The participants (P) were interviewed until data saturation was reached. Member checking was carried out after data analysis, and preliminary findings of the study were discussed with the participants to validate the results. Referential adequacy was ensured by using a voice recorder to capture information obtained from the patient during interviews, and field notes were also taken. Dependability was achieved by the use of an independent coder who dealt with the raw data to develop themes and sub-themes, and a consensus was reached with the researcher for finalisation of themes and sub-themes. Transferability was ensured by thick description of research methodology. Confirmability was ensured by transcribing the recorded information word for word, and the non-verbal cues such as sigh, frowns and head shaking were recorded in brackets of the transcripts to ensure authenticity.

### Ethical considerations

University of Venda Research Ethics Committee provided an ethical clearance to conduct the study (reference number: SHS/16/ PDC/06/1304). The Limpopo Provincial Department of Health (reference number: 4/2/2) granted the permission to access the selected health facilities. The managers of the selected facilities gave permission for the researcher to access participants. Each participant gave informed, written consent before the interviews were conducted and had the right to withdraw from the study at any time without any penalty. Ethical principles of fairness, privacy, confidentiality and anonymity in the study were considered. The researcher also obtained permission from participants for the use of a voice recorder to capture interviews.

## Results

### Demographic data

Twenty-five newly qualified midwives from the five selected hospitals, employed during their 1st year of placement after completion of training were interviewed. The newly qualified midwives shared their experiences with regard to provision of midwifery services during transition from students to qualified midwives. Three themes and four sub-themes emerged during data analysis, as presented in Table 1.

**TABLE 1:** Themes and sub-themes of the study.

Themes	Sub-themes
**Theme 1:** Excessive workload, resulting in physical exhaustion	Labour ward viewed as a negative working environment
**Theme 2:** Roles and responsibilities of newly qualified midwives	Supervisory and teaching roles and responsibilities of newly qualified midwives
	Administrative roles of newly qualified midwives
**Theme 3:** Collegial relationships: A burden on emotional well-being	Negative attitudes of experienced midwives

*Source:* Simane-Netshisaulu, K.G., Maputle, M.S., Netshikweta, M.L. & Shilubane, N.H., 2018, *Transition support programme for newly graduated midwives in Limpopo province, South Africa*, PhD-Thesis, University of Venda, Thohoyandou, South Africa.

### Themes and sub-themes

#### Theme 1: Excessive workload, resulting in physical exhaustion

**Sub-theme 1.1: Labour ward viewed as a negative working environment:** Newly qualified midwives reported bad experiences in the labour ward as a result of a heavy workload leading to physical exhaustion. The newly qualified midwives revealed that they knew that the labour ward was an intense unit to work in but was under the impression that working as a qualified midwife would not be as strenuous. This was supported by the following quotes from participants:

‘The delivery room is so hectic, at times one may even think of removing one’s shoes and walk barefooted. Oh! I am tired of working in the delivery room.’ (P1, 24 years old, female)

‘The situation is very bad, it is more difficult than I thought. I never knew that being a qualified midwife is hard, especially in the labour ward. You are expected to perform like senior staff members, mind you. As a neophyte you are inexperienced.’ (P3, 25 years old, female)

Newly qualified midwives emphasised that they expected to be supported and taken through their transition journey, unfortunately that was not the case as they were always reminded of shortage of staff whenever they asked for assistance, which negatively affected their work performance. As a result of staff shortage, the newly qualified midwives often act as professional nurses in charge of the labour ward. One newly qualified midwife reported that:

‘Besides the business of the delivery room, staff shortage is also a problem. One of the reasons why we are not supported is that experienced midwives are very few because of shortage.’ (P4, 26 years old, male)

#### Theme 2: Roles and responsibilities of newly qualified midwives

**Sub-theme 2.1: Supervisory and teaching roles and responsibilities of newly qualified midwives:** Newly qualified midwives were faced with challenges related to supervision and teaching roles and responsibilities accompanying a new position. Participants expressed conflicting feelings of happiness for their achievement. However, being responsible for students and junior staff members’ learning needs posed challenges to the newly qualified midwives.

One newly qualified midwife reported:

‘I am glad that I successfully made it to be a professional nurse, the problem is the fact that all junior staff members expect that I provide answers to their problems. When I think about that I feel like not coming on duty. Mhhhhhh….. it’s so awkward to be expected to take a supervisory and teaching role.’ (P12, 23 years old, male)

‘One thing that makes me scared is the reality that I must stand as a professional nurse and supervise students as well as patient care. We were never given a chance to decide on patient care during training, our responsibility as students was to carry out instructions from the doctors and professional nurses, but now you are expected to make decisions that affect the patients’ lives. It’s not easy, it’s stressful.’ (P8, 22 years old, female)

The findings revealed that somehow participants still appreciated and wished they were still on training, especially when faced with situations whereby they had to make decisions affecting patients’ lives as well as students on training.

**Sub-theme 2.2: Administrative roles of newly qualified midwives:** Although newly qualified midwives were theoretically trained on professional nurse’s managerial role, they suffered anxiety because of lack of confidence in functioning as unit managers. Control and management of highly scheduled drugs were reported to be alarming and participants needed strong support from experienced midwives. Delegation of duties to members of staff was reported to be a difficult task, especially because graduates are young and consider most of the staff members as their elders. A participant said:

‘When we were students, we were never given any chance to practice managing the unit, but suddenly you are expected to manage the unit including patients, staff members, equipment and supplies. This is not easy. Especially because you don’t feel confident enough to delegate duties to some members of staff.’ (P9, 25 years old, male)

Another newly qualified midwife reported:

‘Procedures such as writing of duty schedules, report writing and giving of reports to the nursing service managers are not easy, especially if you don’t get effective support from the experienced midwives. Another thing that really frustrates me is to attend meetings where you are expected to report about your unit. Oh! That is indeed a problem.’ (P5, 24 years old, female)

Participants expressed challenges regarding attendance of meetings on behalf of the units in which they work. Newly qualified midwives reported a lack of confidence as a challenge regarding report-giving.

#### Theme 3: Collegial relationships: A burden on emotional well-being

**Sub-theme 3.1: Negative attitudes of experienced midwives:** Participants reported that negative comments and a lack of respect from senior midwives affected their performance negatively as they felt humiliated and undermined.

Graduates reported frustration resulting from negative responses from experienced midwives when asking for assistance. Negativity was sometimes displayed through body posture such as ‘keeping quiet when spoken to’ (P22, 23 years old, female). Another newly qualified midwife reported:

‘The relationship between us and some senior members is ok, however, some are unfriendly. The unfriendly ones give bad and demotivating remarks when we seek for assistance. It’s bad.’ (P11, 24 years old, female)

‘What can you do? Because if you seek for assistance, they say you are testing them, if you keep quiet they say you consider yourself as a better person who knows much. What can you do to make them happy?’ (P17, 25 years old, male)

The findings of the study revealed that participants experienced frustrations and anger as some of the senior midwives were not willing to provide assistance when sought. Sometimes participants had to buy favours from their senior colleagues as they knew they were not accepted.

## Discussion

Newly qualified midwives’ experiences in the labour ward were traumatic, resulting in physical exhaustion. This was because of high intensity of the workload, which made labour ward to be perceived as an overwhelming environment as it predisposed newly qualified midwives to high levels of distress and anxiety. This is consistent with the findings by Hobbs (2012) and Lennox and Foureur (2012) as well as Simane-Netshisaulu, Maputle, Netshikweta and Shilubane (2018), whereby participants reported that allocation of midwifery graduates in delivery rooms, who were predisposed to frustration and confusion, were left struggling to cope, as their level of confidence was negatively affected.

Newly qualified midwives expressed dissatisfaction because their senior midwifery colleagues had high expectations from their performance. In some instances, newly qualified midwives had to be in-charge of the delivery rooms. Kitson-Reynolds et al. (2015) shared the same sentiments by reporting that graduates did not want to be allocated in the hospital’s labour ward as they perceived it to be a hectic ward because one midwife would be expected to take care of up to 30 mothers with their newborns within the first few weeks of transition from being a student to a qualified midwife.

The findings revealed that support of newly qualified midwives by senior midwifery colleagues was not effective and shortage of staff was used as a scapegoat for failure to provide effective support. Newly qualified midwives felt a need for working under supervision during their community service, which was not the case as an underresourced working environment interfered with the community service goal of guidance (Ngcobo et al. 2021). The authors further reported that participants felt pressurised as they were expected to work independently without being supervised (Ngcobo et al. 2021).

Graduates did not feel comfortable supervising colleagues as they felt they themselves needed to be supervised. Newly qualified midwives reported that they did not feel competent and confident regarding performance of a teaching role. The findings revealed that newly qualified midwives got theoretical background on managerial tasks; however, clinical areas of practice on management of scheduled drugs, problem-solving, decision making and management of material resources were not adequately addressed during students’ training. Newly qualified midwives reported feeling overwhelmed and inadequate in clinical management of the unit.

Literature confirmed that newly qualified midwives reported their inability to carry out their administrative role, resulting in poor self-worth; therefore, recommended that a structured support programme be put in place to facilitate effective transition from being students to becoming qualified midwives (Cummins Denney-Wilson & Homer 2016; Netshisaulu & Maputle 2018; Ngcobo et al. 2021; Power 2016).

Midwifery graduates expressed anger and frustration resulting from negative relationship displayed by their senior colleagues. A series of negative comments passed by experienced midwives, whenever graduates pleading for assistance was reported. Graduates felt offended and humiliated because of negative attitudes and lack of respect displayed towards them by experienced midwives. Sometimes graduates had to go out of their way trying to please experienced midwives to buy harmonious relationships with them as they felt they did not belong to the team. This was because graduates felt unwelcomed leading to a feeling of unworthiness and low self-esteem, which negatively affected their confidence. Griffiths et al. (2019) shared the same sentiments when they reported that newly qualified midwives experienced rejection and isolation as they were ignored when seeking help, which caused distress especially in front of the patients and graduates interpreted the behaviour as being disrespectful (Javanmard et al. 2019; Kensington et al. 2016). Provision of positive support by experienced midwives made newly qualified midwives to feel valued when treated positively as part of the team and having responsibility for practice, which contributed greatly to learning and boosted their confidence, thus, improving their performance (Bradshaw, Tighe & Doody 2018; Simane-Netshisaulu & Maputle 2021). Newly qualified midwives were not satisfied about the type of relationship they had with their senior colleagues, which made them suffer from a feeling of lack of identity and sense of belonging to the team, thus negatively impacting the type of midwifery services graduates provided (Kensington et al. 2016). Graduates felt they would have been supported effectively if the relationship with their senior colleagues was positive (Wain 2017). A supportive environment in which positive midwife-to-midwife relationships exist is vital in ensuring a successful transitional journey of newly qualified midwives.

## Conclusion

Newly qualified midwives found transition process stressful and exhausting because of unfavourable working conditions in the labour ward. Roles regarding unit management posed challenges to newly qualified midwives, as they were not confident to perform them. Negative collegial relationship and disrespect displayed by experienced midwives had negative impact on graduates’ performance. It is therefore recommended that shortage of staff be addressed by managers, and this may assist in curbing the issue of heavy workload. Mangers should devise strategies to effectively support newly qualified midwives as this may assist in improving graduates’ performance, thus increasing productivity.

## References

[CIT0001] Black, S.E., 2018, ‘Does preceptorship support newly qualified midwives to become confident practitioners?’, *British Journal of Midwifery* 26(12), 806–811. 10.12968/bjom.2018.26.12.806

[CIT0002] Bradshaw, C., Tighe, S.M. & Doody, O., 2018, ‘Midwifery students’ experiences of their clinical internship: A qualitative descriptive study’, *Nurse Education Today* 68, 213–217. 10.1016/j.nedt.2018.06.019 29966883

[CIT0003] Creswell, J.W., 2016, *Research design: Qualitative, quantitative and mix methods approaches*, Sage, Thousand Oaks, CA.

[CIT0004] Cummins, A.M., Denney-Wilson, E. & Homer, C.S.E., 2016, ‘The mentoring experiences of new graduate midwives working in midwifery continuity of care models in Australia’, *Nurse Education in Practice* 12(2), 1–6. 10.1016/j.nepr.2016.01.003 26830916

[CIT0005] De Vos, A.S., Strydom, H., Fouche, C.B. & Delport, C.S.L., 2013, *Research at grass roots: For the social sciences and human service professions*, Van Schaik, Pretoria.

[CIT0006] Dixon, L., Calvert, S., Tumilty, E., Kensington, M., Gray, E., Campbell, N. et al., 2015, ‘Supporting New Zealand graduate midwives to stay in the profession: An evaluation of the midwifery first year of practice programme’, *Midwifery* 12(4), 102–114. 10.1016/j.midw.2015.02.010 25819705

[CIT0007] Duchscher, J.E.B., 2009, ‘Transition shock: The initial stage of role adaptation for newly graduated registered nurses’, *Journal of Advanced Nursing* 65(5), 1103–1113. 10.1111/j.1365-2648.2008.04898.x 19183235

[CIT0008] Etikan, I. & Bala, K., 2017, ‘Sampling and sampling methods’, *Biometrics & Biostatistics International Journal* 5(6), 00149. 10.15406/bbij.2017.05.00149

[CIT0009] Griffiths, M., Fenwick, J., Carter, A.G., Sidebotham, M. & Gamble, J., 2019, ‘Midwives transition to practice: Expectations and experiences’, *Nurse Education in Practice* 41(10), 26–41. 10.1016/j.nepr.2019.102641 31630046

[CIT0010] Hobbs, J.A., 2012, ‘Newly qualified midwives’ transition to qualified status and role: Assimilating the “habitus” or reshaping it?’, *Midwifery* 28(4), 391–399. 10.1016/j.midw.2011.04.007 22119403

[CIT0011] Javanmard, M., Steen, M., Vernon, R. & Cooper, M., 2019, ‘Transition experiences of internationally qualified midwives practicing midwifery in Australia’, *Women and Birth* 8(6), 1–11. 10.1016/j.wombi.2019.05.002 31151889

[CIT0012] Kensington, M., Campbell, N., Gray, E., Dixon, L., Tumilty, E., Pairman, S. et al., 2016, ‘New Zealand’s midwifery profession: Embracing graduate midwives’ transition to practice’, *New Zealand College of Midwives Journal* 52(4), 20–25. 10.12784/nzcomjnl52.2016.3.20-25

[CIT0013] Kitson-Reynolds, E., Ferns, P. & Trenerry, A., 2015, ‘Transition to midwifery: Collaborative working between university and maternity services’, *British Journal of Midwifery* 23(7), 510–515. 10.12968/bjom.2015.23.7.510

[CIT0014] Lennox, S. & Foureur, M., 2012, ‘Developmental mentoring: New graduates’ confidence grows when their needs shape the relationship’, *New Zealand College of Midwives Journal* 46(11), 26–31.

[CIT0015] Lewis, S. & McGowan, B., 2015, ‘Newly qualified nurse’s experiences of preceptorship’, *British Journal of Nurses* 24(1), 40–43. 10.12968/bjon.2015.24.1.40 25541875

[CIT0016] Ndaba, B.J., 2013, ‘Lived experiences of newly qualified professional nurses doing community service in midwifery section in one Gauteng hospital’, *Curationis* 23(5), 15–22.

[CIT0017] Netshisaulu, K.G. & Maputle, M.S., 2018, ‘Expected clinical competence from midwifery graduates during community service placement in Limpopo province, South Africa’, *Health SA Gesondheid* 23, 1–7. 10.4102/hsag.v23i0.1166 PMC6917449 31934392

[CIT0018] Ngcobo, A., Baloyi, O.B. & Jarvis, M.A., 2021, ‘Newly qualified midwives’ perceptions of their level of midwifery clinical competence during community service in KwaZulu-Natal, South Africa’, *Health SA Gesondheid* 26, a1670. 10.4102/hsag.v26i0.1670 PMC8603134 34858648

[CIT0019] Odland, L.H., Sneltvedt, T. & Sörlie, V., 2014, ‘Responsible but unprepared: Experiences of newly educated nurses in hospital care’, *Nurse Education Practice* 14(5), 538–543. 10.1016/j.nepr.2014.05.005 24882569

[CIT0020] Power, A., 2016, ‘Midwifery in the 21st century: Are students prepared for the challenge?’, *British Journal of Midwifery* 24(1), 66–68. 10.12968/bjom.2016.24.1.66

[CIT0021] Reynolds, E.K., Cluett, E. & Le-May, A., 2014, ‘Fairy tale, midwifery fact or fiction: The lived experiences of newly qualified midwives’, *British Journal of Midwifery* 22(9), 660–668. 10.12968/bjom.2014.22.9.660

[CIT0022] Royal College of Midwives, 2014, *High quality midwifery care*, RCM, London.

[CIT0023] Simane-Netshisaulu, K.G. & Maputle, M.S., 2019, ‘Clinical practice of midwifery graduates during community service placement, Limpopo Province South Africa’, *Global Journal of Health Science* 11(10), 97–104. 10.5539/gjhs.v11n10p97 PMC6917449 31934392

[CIT0024] Simane-Netshisaulu, K.G., Maputle, M.S., Netshikweta, M.L. & Shilubane, N.H., 2018, *Transition support programme for newly graduated midwives in Limpopo province, South Africa*, PhD-Thesis, University of Venda, Thohoyandou, South Africa.

[CIT0025] Simane-Netshisaulu, K.G. & Maputle, S.M., 2021, ‘Exploring supportive relationship provided to newly qualified midwives during transition period in Limpopo province, South Africa’, *African Journal of Reproductive Health* 25(5), 105–113. 10.29063/ajrh2021/v25i5.11 37585864

[CIT0026] South African Nursing Council (SANC), 1992, *Regulations relating to the approval of and the minimum requirements for the education and training of a nurse (general, psychiatric and community) and midwife leading to registration, (R425 of February 1985)*, Government Printer, Pretoria.

[CIT0027] Van Der Putten, D., 2008, ‘The lived experience of newly qualified midwives: A qualitative study’, *British Journal of Midwifery* 16(6), 348–359. 10.12968/bjom.2008.16.6.29592

[CIT0028] Wain, A., 2017, ‘Examining the lived experiences of newly qualified midwives during their preceptorship’, *British Journal of Midwifery* 25(7), 451–457. 10.12968/bjom.2017.25.7.451

